# Identification of MupP as a New Peptidoglycan Recycling Factor and Antibiotic Resistance Determinant in *Pseudomonas aeruginosa*

**DOI:** 10.1128/mBio.00102-17

**Published:** 2017-03-28

**Authors:** Coralie Fumeaux, Thomas G. Bernhardt

**Affiliations:** Department of Microbiology and Immunobiology, Harvard Medical School, Boston, USA; University of Chicago

## Abstract

Peptidoglycan (PG) is an essential cross-linked polymer that surrounds most bacterial cells to prevent osmotic rupture of the cytoplasmic membrane. Its synthesis relies on penicillin-binding proteins, the targets of beta-lactam antibiotics. Many Gram-negative bacteria, including the opportunistic pathogen *Pseudomonas aeruginosa*, are resistant to beta-lactams because of a chromosomally encoded beta-lactamase called AmpC. In *P. aeruginosa*, expression of the *ampC* gene is tightly regulated and its induction is linked to cell wall stress. We reasoned that a reporter gene fusion to the *ampC* promoter would allow us to identify mutants defective in maintaining cell wall homeostasis and thereby uncover new factors involved in the process. A library of transposon-mutagenized *P. aeruginosa* was therefore screened for mutants with elevated *ampC* promoter activity. As an indication that the screen was working as expected, mutants with transposons disrupting the *dacB* gene were isolated. Defects in DacB have previously been implicated in *ampC* induction and clinical resistance to beta-lactam antibiotics. The screen also uncovered *murU* and *PA3172* mutants that, upon further characterization, displayed nearly identical drug resistance and sensitivity profiles. We present genetic evidence that *PA3172*, renamed *mupP*, encodes the missing phosphatase predicted to function in the MurU PG recycling pathway that is widely distributed among Gram-negative bacteria.

## IMPORTANCE

The cell wall biogenesis pathway is the target of many of our best antibiotics, including penicillin and related beta-lactam drugs. Resistance to these therapies is on the rise, particularly among Gram-negative species like *Pseudomonas aeruginosa*, a problematic opportunistic pathogen. To better understand how these organisms resist cell wall-targeting antibiotics, we screened for *P. aeruginosa* mutants defective in maintaining cell wall homeostasis. The screen identified a new factor, called MupP, involved in the recycling of cell wall turnover products. Characterization of MupP and other components of the pathway revealed that cell wall recycling plays important roles in both the resistance and the sensitivity of *P. aeruginosa* to cell wall-targeting antibiotics.

## INTRODUCTION

*Pseudomonas aeruginosa* is an opportunistic Gram-negative pathogen capable of growth in diverse environments (1). In hospitals, it causes a number of serious infections (2, 3). The key drugs in our arsenal for treating these infections are the beta-lactam antibiotics, including cephalosporins, monobactams, and carbapenems, which target the biogenesis of the peptidoglycan (PG) cell wall (4). Resistance to these antibiotics is on the rise among Gram-negative bacteria like *P. aeruginosa* and is often associated with multidrug resistance phenotypes. A frequent mechanism of resistance to beta-lactams is overproduction of the chromosomally encoded beta-lactamase called AmpC, which inactivates penicillins, cephalosporins, and monobactams (5–8).

AmpC is a broadly distributed group I, class C cephalosporinase produced by most *Enterobacteriaceae* family members and many nonfermenting Gram-negative bacilli in addition to *P. aeruginosa* (9). In the absence of stress, AmpC production is relatively low in wild-type strains (10). However, in the presence of certain beta-lactams, such as cefoxitin (Fox) and imipenem (beta-lactamase inducers), *ampC* expression is highly activated (10). Although they are sensitive to hydrolysis by AmpC, antipseudomonal penicillins like piperacillin (Pip) and cephalosporins like ceftazidime (Caz) are effective because they avoid *ampC* induction (11). However, mutants defective in *ampC* regulation that constitutively produce high levels of beta-lactamase have been isolated in the clinic and can cause failures of antimicrobial therapy (7, 12–16).

**FIG 1:**
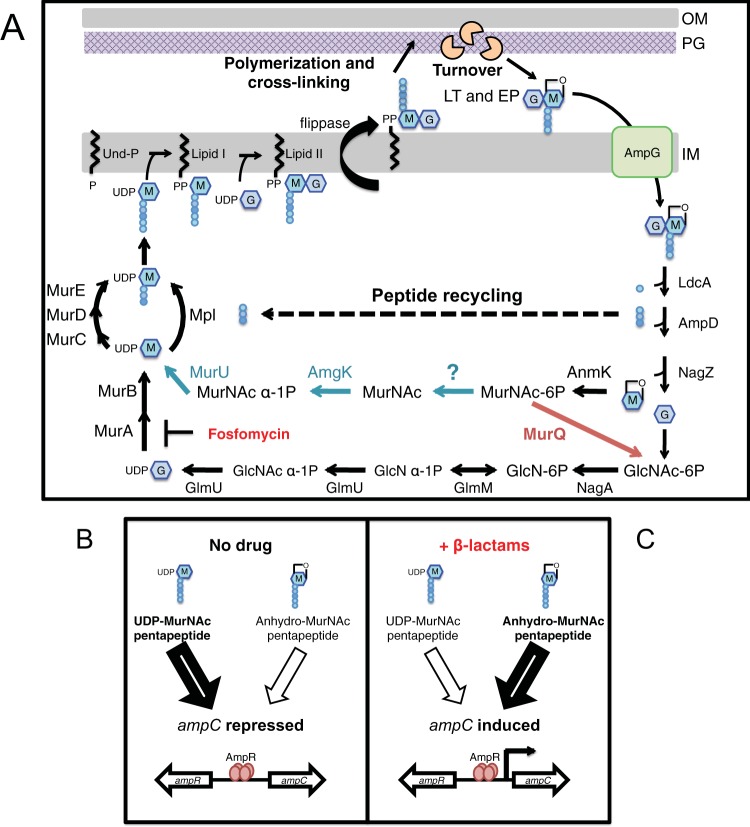
Simplified pathways for PG synthesis and recycling and the link to *ampC* regulation. (A) The PG matrix consists of glycan chains with the repeating unit of MurNAc (M) and GlcNAc (G). Attached to the MurNAc sugars is a pep5 (l-Ala-γ-d-Glu-*meso*-diaminopimelic acid-d-Ala-d-Ala, colored circles) used to form cross-links between adjacent glycans. PG synthesis starts in the cytoplasm, is continued by the generation of lipid-linked precursors, and ends with the polymerization and cross-linking reactions at the membrane surface to build PG. The matrix is also subject to degradation by LTs and EPs to generate anhMurNAc-containing turnover products, which are recycled. The names of the general recycling enzymes present in both *E. coli* and *P. aeruginosa* are black. The proteins found uniquely in *E. coli* and in *P. aeruginosa* are red and blue, respectively. See the text for details. (B) Under normal conditions (no drug, left side), the PG precursor UDP-MurNAc-pep5 binds to AmpR and causes repression of *ampC* transcription (35, 36). During beta-lactam stress (right side), PG cross-linking is blocked and turnover is elevated (37). This imbalance causes accumulation of anhMurNAc-pep5 and GlcNAc-anhMurNAc-pep5 in the cytoplasm. The accumulated anhydro-muropeptides are thought to competitively displace UDP-MurNAc-pep5 from AmpR and convert it into an activator of *ampC* transcription (10, 38, 40, 41).

The mechanism of *ampC* regulation is intimately connected to the PG synthesis and recycling pathways (Fig. 1) (17). PG synthesis begins in the cytoplasm with the formation of UDP–*N*-acetylmuramic acid (UDP-MurNAc) from UDP–*N*-acetylglucosamine (UDP-GlcNAc) through the action of the enzymes MurA and MurB. A pentapeptide (pep5) is added to UDP-MurNAc in several steps, forming UDP-MurNAc-pep5. The phospho-MurNAc-pep5 moiety of this intermediate is then transferred to the lipid carrier undecaprenol phosphate (Und-P), forming lipid I. GlcNAc from UDP-GlcNAc is then added to form lipid II, which is the final precursor and contains the MurNAc-pep5-GlcNAc monomeric unit of PG. After lipid II is translocated (18) to expose the disaccharide-peptide on the outer surface of the cytoplasmic membrane, it is polymerized and cross-linked into the PG layer by penicillin-binding proteins (PBPs) (19) and SEDS family proteins (20) to expand the existing matrix.

Far from being inert, the PG layer is constantly remodeled during cell growth. Roughly 40% of the PG layer is turned over per generation in *Escherichia coli* (21). The liberated fragments are primarily generated by the action of endopeptidases (EPs) that cleave the peptide cross-links and lytic transglycosylases (LTs) that cleave the sugar backbone. Rather than hydrolyzing the glycans, LTs promote the formation of 1,6-anhydro linkages in MurNAc such that the main PG degradation products released from the matrix are GlcNAc-1,6-anhMurNAc peptides (21) (Fig. 1). These anhydro-muropeptides are subsequently transported into the cytoplasm by the permease AmpG (22) and possibly AmpP in *P. aeruginosa* (23), where they are further broken down into their basic components by a succession of enzymes (21, 24) (Fig. 1). The glycosidase NagZ removes the GlcNAc moiety (25, 26), and the amidase AmpD removes the stem peptide from the NagZ-processed product or the incoming disaccharide (27, 28). The released peptides are further processed to tripeptides by the l,d-carboxypeptidase LdcA and reattached to UDP-MurNAc for recycling by Mpl (29, 30) (Fig. 1). Recycling of the PG sugars is carried out by one of two possible pathways in Gram-negative bacteria (Fig. 1). The first pathway was discovered in *E. coli* and ultimately converts GlcNAc and 1,6-anhMurNAc to glucosamine-1-phosphate (GlcN-1P) for the regeneration of UDP-GlcNAc by the *de novo* biosynthesis pathway involving GlmU (21, 31, 32) (Fig. 1). The second pathway was discovered recently and is more broadly conserved among Gram-negative bacteria, including *P. aeruginosa* (33). It uses the enzymes AmgK and MurU to more directly convert 1,6-anhMurNAc back to UDP-MurNAc, thus bypassing *de novo* biosynthesis (33, 34).

The main regulator of *ampC* expression is AmpR. In nonstressed cells, it associates with the PG precursor UDP-MurNAc-pep5 and functions as a repressor (35, 36). Beta-lactams inhibit PG cross-linking by the PBPs, causing the formation of uncross-linked glycans that are rapidly degraded by LTs into turnover products (37). The resulting accumulation of anhydro-muropeptides in the cytoplasm is thought to compete with UDP-MurNAc-pep5 for binding to AmpR and convert the regulator into an activator of *ampC* transcription (10, 38–41). Following AmpC production and export to the periplasm, the beta-lactam molecules are inactivated by hydrolysis and homeostasis is restored, eventually resulting in a decrease in cytoplasmic anhydro-muropeptide levels and repression of *ampC* (42).

Because it functions as a key sensor of PG homeostasis, we reasoned that an *ampC* promoter fusion to *lacZ* might serve as a useful tool to identify new *P. aeruginosa* factors involved in cell wall synthesis, repair, and recycling. To this end, we mutagenized a strain encoding a chromosomally integrated P_*ampC*_::*lacZ* fusion (43) with a transposon and plated the resulting mutant library on plates containing X-Gal (5-bromo-4-chloro-3-indolyl-β-d-galactopyranoside). Colonies displaying increased blue color, indicative of P_*ampC*_::*lacZ* induction, were isolated, and the locations of transposon insertions in these isolates were mapped. As an indication that the screen was working as expected, mutants with transposons disrupting *dacB* were isolated. DacB defects have previously been implicated in *ampC* induction and clinical resistance to beta-lactam antibiotics (7, 14). The screen also uncovered *murU* and *PA3172* mutants that, upon further characterization, displayed nearly identical drug resistance and sensitivity profiles. We present genetic evidence that *PA3172*, renamed *mupP*, encodes the missing phosphatase enzyme previously predicted (33) to function in the broadly distributed MurU pathway for PG recycling. Biochemical results in a parallel study by the Mayer group support this designation (44).

## RESULTS

### Identification of transposon mutants that induce *ampC* expression.

To identify new factors involved in PG homeostasis, recycling, and remodeling, we took advantage of the connection between *ampC* induction and cell wall stress (10, 28). A strain bearing a P_*ampC*_::*lacZ* expression construct at the *attB* locus (43) was generated to search for mutants displaying a constitutive *ampC* induction phenotype. To test the activity of the reporter and its responsiveness to cell wall defects, we deleted the *dacB* gene in the reporter strain. DacB is a cell wall carboxypeptidase that trims the peptide within PG (45, 46). Its inactivation was previously shown to cause constitutive expression of *ampC* (7). As expected, the Δ*dacB* mutant reporter strain formed dark blue colonies on LB agar containing X-Gal. Reporter activity in this background was abolished upon inactivation of the AmpG permease, indicating that P_*ampC*_::*lacZ* induction in the Δ*dacB* background requires the import of PG turnover products, as has been shown previously for the native *ampC* locus (47). On the basis of its behavior in these mutant backgrounds, we concluded that the P_*ampC*_::*lacZ* reporter strain was functional and appropriate for use in screening for cell wall homeostasis mutants.

**FIG 2:**
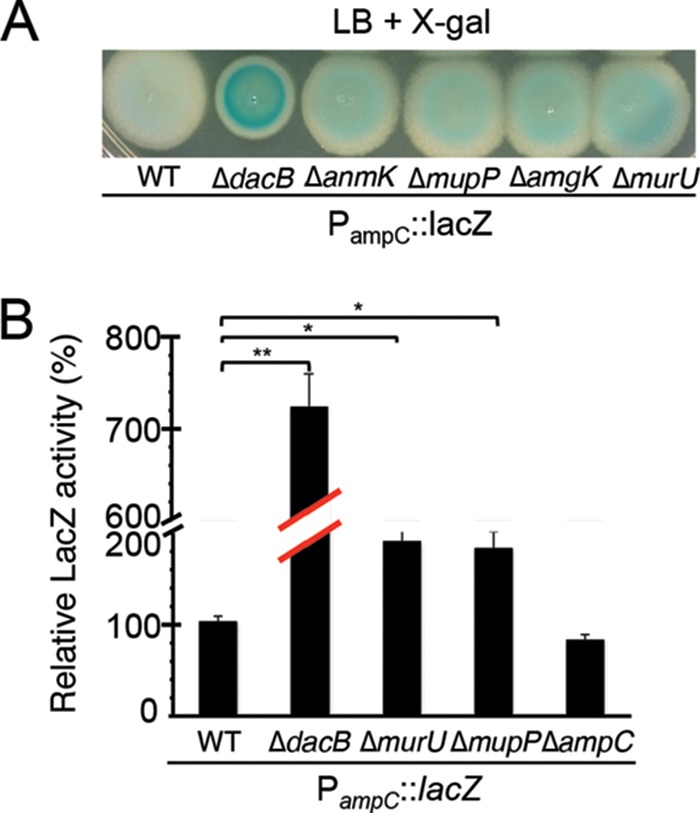
P_*ampC*_::*lacZ* expression in *mupP* and *murU* deletion strains. (A) Cultures (5 µl) of strains PAO1 (wild type [WT]), CF268 (Δ*dacB* mutant), CF706 (Δ*anmK* mutant), CF594 (Δ*mupP* mutant), CF600 (Δ*amgK* mutant), and CF485 (Δ*murU* mutant) containing the P_*ampC*_::*lacZ* reporter were spotted onto LB agar containing X-Gal (50 µg/ml), grown overnight at 30°C, and photographed. (B) β-Galactosidase activity was measured in liquid cultures of the strains indicated. The activity in the wild-type strain was set at 100%, and the activity in the other strains is reported relative to wild-type activity. Results shown are the averages of three assays with two biological replicates per strain, and the error bars represent the standard deviation. *, *P* < 0.01; **, *P* < 0.0001 (compared to wild-type expression, as determined by Welch’s unequal-variance *t* test).

Cells of the reporter strain CF263 (PAO1 P_*ampC*_::*lacZ*) were mutagenized with a transposon carrying a tetracycline (Tet) resistance cassette that was delivered by conjugation from *E. coli*. The resulting mutant library was then plated on agar containing X-Gal to identify constitutive P_*ampC*_ mutants. Colonies displaying increased blue color, indicative of *lacZ* induction, arose at a frequency of approximately 10^−5^. Following purification, isolates were grown in liquid medium to measure beta-galactosidase activity relative to that of the parental strain. The transposon insertion sites were then mapped for strains confirmed to have elevated *lacZ* expression. As an indication that the screen was working as expected, two mutants were isolated that each possessed a different insertion in the *dacB* gene. In addition to these strongly induced alleles, we also isolated mutants that formed light blue colonies on X-Gal agar and had mildly elevated beta-galactosidase activity (Fig. 2). Mapping revealed that these isolates had transposon insertions in the *murU* and *PA3172* genes. The absence of *ampD* mutants (14) among our isolates indicates that the screen is not yet saturated and further screening should yield additional mutants that activate the *ampC* reporter.

MurU is an α-1-phosphate uridyl transferase that converts MurNAc-1P to UDP-MurNAc in the *Pseudomonas* PG recycling pathway (33) (Fig. 1). The *PA3172* gene is annotated as encoding a phosphoglycolate phosphatase, and its product was found to possess phosphatase activity against small-molecule substrates with a phosphate moiety (48). This activity of PA3172 was intriguing because a phosphatase was previously predicted to function in the MurU PG recycling pathway but has remained unidentified (33) (Fig. 1). Because of its biochemical activity and the similar P_*ampC*_::*lacZ* induction phenotypes displayed by mutants with *murU* and *PA3172* inactivated, we hypothesized that *PA3172* may encode the missing recycling phosphatase. Results presented below and those from a parallel study by the Mayer group (44) support this hypothesis. We therefore have renamed the *PA3172* gene *mupP* for MurNAc-6P phosphatase.

### Deletion of *mupP* increases *ampC* expression and promotes beta-lactam resistance similar to other PG recycling mutants.

**FIG 3:**
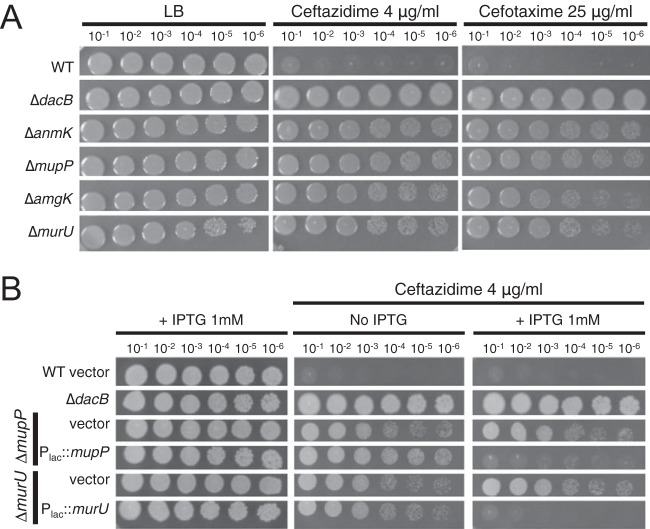
Beta-lactam resistance of strains with PG recycling factors deleted. (A) Cultures of strains PAO1 (wild type [WT]), CF155 (Δ*dacB* mutant), CF550 (Δ*anmK* mutant), CF592 (Δ*mupP* mutant), CF596 (Δ*amgK* mutant), and CF488 (Δ*murU* mutant) were serially diluted, and 5 µl of each dilution was spotted onto LB agar supplemented with Caz (4 µg/ml) or Ctx (25 µg/ml), as indicated. The Caz and Ctx MICs determined by agar dilution were 2.5 and 25 µg/ml for the wild type and 5 and 30 µg/ml for the recycling mutants, respectively. An increase in the MICs for the recycling mutants was not observed in liquid medium. (B) Cultures of CF732 (PAO1 [empty]), CF155 (Δ*dacB* mutant), CF521 (Δ*mupP* [empty]), CF505 (Δ*mupP* [P_*lac*_::*mupP*]), CF517 (Δ*murU* [empty]), and CF519 (Δ*murU* [P_*lac*_::*murU*]) were serially diluted and plated on LB agar supplemented with IPTG (1 mM), Caz (4 µg/ml), or both, as indicated. Expression constructs were integrated at the *att*Tn*7* locus.

**FIG 4:**
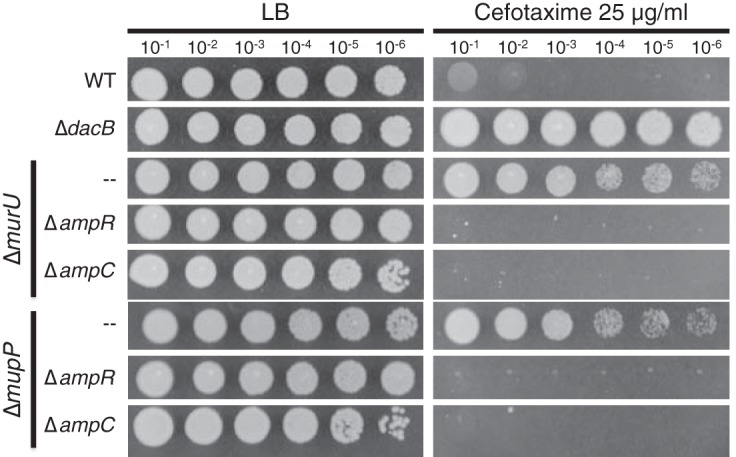
AmpR and AmpC are required for the beta-lactam resistance phenotype of Δ*murU* and Δ*mupP* mutant strains. Cultures of strains PAO1 (wild type [WT]), CF155 (Δ*dacB*) mutant, CF488 (Δ*murU* mutant), CF690 (Δ*murU* Δ*ampC* mutant), CF608 (Δ*murU* Δ*ampR* mutant), CF592 (Δ*mupP* mutant), CF692 (Δ*mupP*Δ*ampC* mutant), and CF647 (Δ*mupP*Δ*ampR* mutant) were serially diluted, and 5 µl of each dilution was spotted onto LB agar with or without Ctx (25 µg/ml), as indicated.

**FIG 5:**
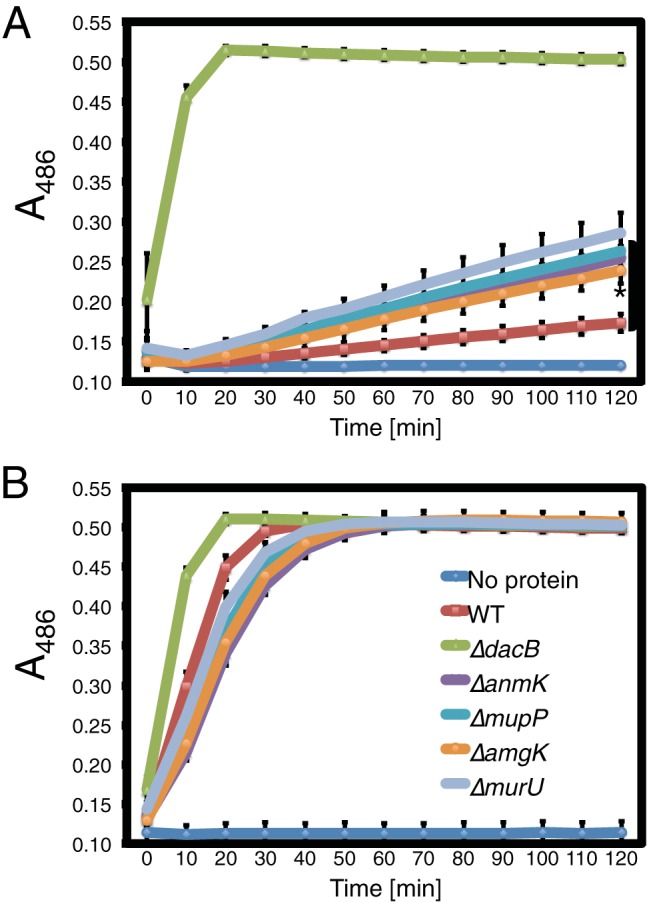
AmpC activity in strains with PG recycling factors deleted. Assay of nitrocefin hydrolysis by cells of PAO1 (wild type [WT]), CF155 (Δ*dacB* mutant), CF550 (Δ*anmK* mutant), CF592 (Δ*mupP* mutant), CF596 (Δ*amgK* mutant), and CF488 (Δ*murU* mutant) grown in LB (A) or LB supplemented with 50 µg/ml Fox (B). The Δ*dacB* mutant served as the positive control and has highly elevated basal AmpC activity, while the recycling mutants have slightly increased activity compared to that of the wild type (PAO1). BSA and the no-protein control have no detectable AmpC activity. Data are the mean of three independent assays each for two biological replicates with the error bars indicating the standard error. *, *P* value < 0.01 compared to wild-type AmpC activity, as determined by Welch’s unequal-variance *t* test.

To confirm their involvement in *ampC* overexpression, in-frame deletions of *murU* and *mupP* were generated in the reporter strain along with deletions in genes coding for other members of the MurU recycling pathway (*anmK* and *amgK*). When these mutants were spotted onto agar containing X-Gal, they gave rise to zones of growth with a light blue color relative to wild-type or Δ*dacB* mutant cells, which appeared white or dark blue, respectively (Fig. 2A). Quantification of beta-galactosidase activity confirmed that mutants defective for *mupP* displayed a similar level of *lacZ* expression as a Δ*murU* mutant strain (Fig. 2B). To monitor the effects of these mutations on native *ampC* induction, the set of deletions in *mupP* and recycling genes was also generated in an otherwise wild-type background. The deletion strains all showed elevated resistance to the antipseudomonal beta-lactams Caz and cefotaxime (Ctx), with resistance being intermediate compared to that of a Δ*dacB* mutant (Fig. 3A). Normal beta-lactam sensitivity was restored to Δ*murU* and Δ*mupP* mutant cells by the expression of the corresponding gene from a plasmid (Fig. 3B), indicating that the phenotype was caused by the inactivation of MurU or MupP and was not an effect of the deletions on the expression of nearby genes. Elevated drug resistance in Δ*murU* and Δ*mupP* mutant cells was dependent on *ampC* and its transcriptional regulator *ampR* (Fig. 4), consistent with resistance arising from *ampC* induction. Finally, *ampC* induction in the recycling mutants was confirmed by directly measuring basal levels of AmpC enzymatic activity by using the reporter substrate nitrocefin (Fig. 5). Notably, inactivation of MupP yielded a level of AmpC activity in cell extracts equivalent to that of strains with defects in the known recycling enzymes MurU, AnmK, and AmgK (Fig. 5A). These strains also retained the ability to induce high levels of AmpC production in response to treatment with the strong inducer Fox (Fig. 5B). As expected from the intermediate drug resistance phenotype, the level of induction of the recycling-defective strains was much less than that of the highly resistant Δ*dacB* mutant. We conclude that mutants with the MurU recycling pathway disrupted have elevated beta-lactam resistance because of *ampC* induction and that mutants with defects in MupP share this phenotype.

### MupP-defective strains are Fos hypersensitive.

**FIG 6:**
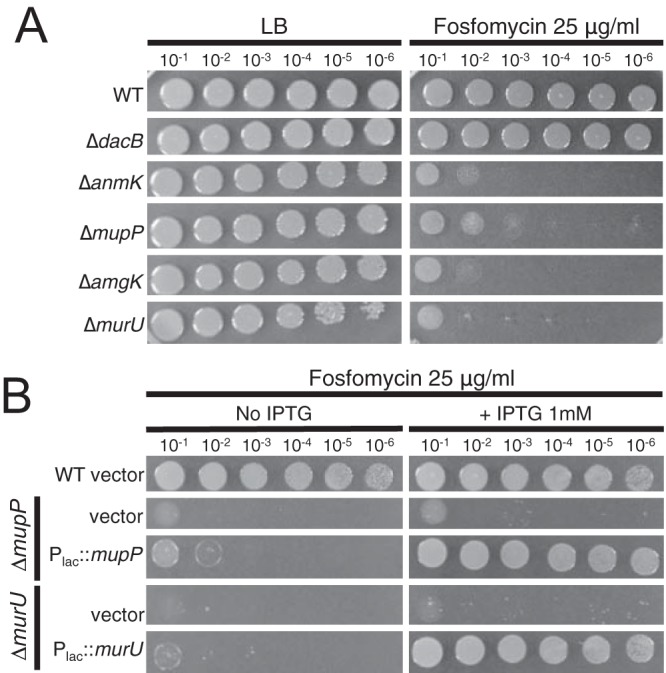
Fos sensitivity of a Δ*mupP* mutant. (A) Cultures of strains PAO1 (wild type [WT]), CF155 (Δ*dacB* mutant), CF550 (Δ*anmK* mutant), CF592 (Δ*mupP* mutant), CF596 (Δ*amgK* mutant), and CF488 (Δ*murU* mutant) were serially diluted, and 5 µl of each dilution was spotted onto LB agar with or without Fos (25 µg/ml), as indicated. The Fos MIC was determined by broth dilution and is >40 µg/ml for the wild type and 15 µg/ml for the recycling mutants, respectively. (B) Cultures of CF732 (PAO1[empty]), CF521 (Δ*mupP* [empty]), CF505 (Δ*mupP* [P_*lac*_::*mupP*]), CF517 (Δ*murU* [empty]), and CF519 (Δ*murU* [P_*lac*_::*murU*]) were serially diluted on LB agar as described for panel A. LB agar was supplemented with 1 mM IPTG, Fos (25 µg/ml), or both, as indicated. Expression constructs were integrated at the *att*Tn*7* locus.

Strains with the recycling gene *murU*, *amgK* or *anmK* inactivated were previously shown to be hypersensitive to the antibiotic fosfomycin (Fos) (33, 34). This drug targets MurA activity and thus blocks the conversion of UDP-GlcNAc into UDP-MurNAc as part of the *de novo* PG precursor synthesis pathway (Fig. 1) (49). A functional MurU pathway bypasses MurA in the conversion of cell wall turnover products into UDP-MurNAc (Fig. 1). It therefore reduces the need for MurA activity, thereby increasing Fos resistance. We reasoned that if MupP is indeed part of the MurU pathway, its inactivation should also result in Fos hypersensitivity. Plating of serial dilutions of Δ*mupP* mutant cells on LB agar with or without Fos revealed a hypersensitivity phenotype that mimicked that of mutants with other components of the MurU pathway deleted (Fig. 6A). As with a *murU* mutant, normal Fos resistance was restored to the Δ*mupP* mutant strain by expression of the *mupP* gene in *trans* from a plasmid (Fig. 6B). This result reinforces the phenotypic similarity of Δ*mupP* mutant cells and mutants with changes in known components of the MurU recycling pathway.

### Expression of *mupP* allows reconstitution of the full MurU pathway in *E. coli*.

**FIG 7:**
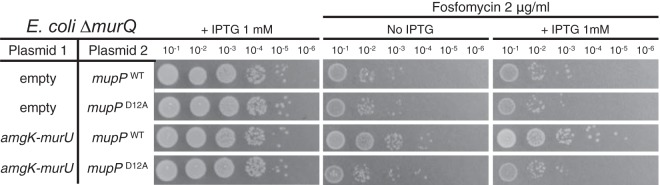
Reconstitution of the complete MurU pathway in *E. coli*. *E. coli* strain CF752 (MG1655 Δ*murQ*) harboring pUC18 (vector) or pCF436 (P_*lac*_::*amgK*-*murU*) along with the compatible vector pCF826 (P_*lac*_::*mupP*) or pCF836 (P_*lac*_::*mupP*[D12A]), as indicated, was serially diluted, and 5 µl of each dilution was spotted onto LB agar supplemented with Fos (2 µg/ml), IPTG (100 µM), or both, as indicated. WT, wild type.

**FIG 8:**
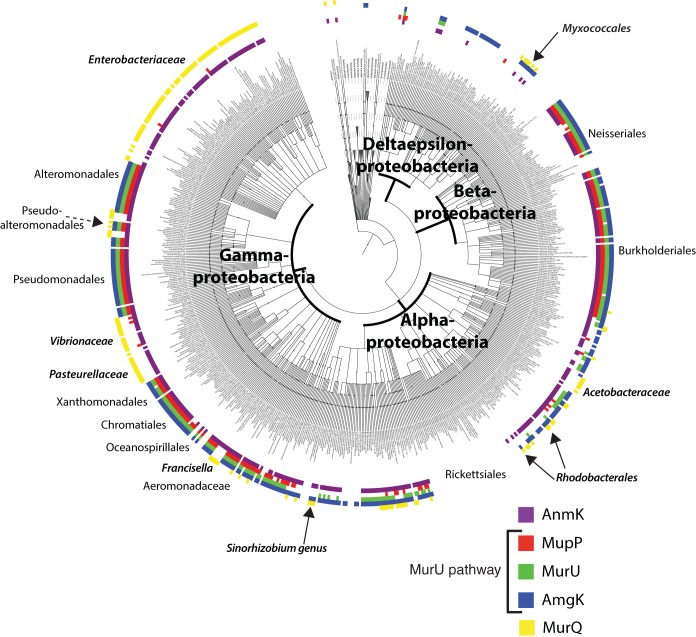
Phylogenetic tree showing AnmK, MupP, AmgK, and MurU protein occurrence and co-conservation. The phylogenetic tree shown was constructed with iTOL (55) and a diversity set of 1,773 strains. The names of the relevant bacterial classes, orders, or families are indicated. The presence of MupP or other PG recycling enzymes (33) in a given species is indicated by the colored regions at the outer edge of the tree and the legend at the lower right.

*E. coli* lacks the MurU pathway and is therefore relatively sensitive to Fos. Instead, it uses the MurQ enzyme to convert MurNAc-6P to GlcNAc-6P for reentry into the *de novo* pathway (Fig. 1). In a Δ*murQ* mutant, MurNAc recycling is blocked at MurNAc-6P. The Mayer group was previously able to partially reconstitute the *P. putida* MurU pathway in an *E. coli* Δ*murQ* mutant, as assessed by increased Fos resistance (33). They did so by expressing *amgK* and *murU* from a plasmid. Because the MurNAc-6P phosphatase remained unidentified at the time, Fos resistance was only restored by supplying MurNAc in the medium for uptake and entry into the pathway. This result suggested that the *E. coli* Δ*murQ* mutant cells were unable to process endogenous MurNAc-6P for use in the recycling pathway by *amgK* and *murU*. Thus, if MupP is indeed the MurNAc-6P phosphatase in the MurU pathway, coexpression of *mupP* with *amgK* and *murU* in *E. coli* Δ*murQ* mutant cells should result in increased Fos resistance without the need for externally added MurNAc. Indeed, expression of wild-type *mupP* in conjunction with *amgK* and *murU* promoted increased Fos resistance to *E. coli* Δ*murQ* mutant cells. Increased resistance was not observed when *mupP* was expressed alone or when a predicted MupP catalytic mutant protein, MupP(D12A) (48), was produced in tandem with AmgK and MurU (Fig. 7). On the basis of these results and the similar phenotypes displayed by *mupP* mutants and mutants defective in PG recycling, we conclude that MupP is the missing phosphatase acting in the MurU pathway. Consistent with this conclusion, MupP is co-conserved with AmgK and MurU in a range of proteobacteria but absent in others like the enterobacteria that lack the MurU pathway (Fig. 8).

## DISCUSSION

Many Gram-negative bacteria encode an inducible AmpC beta-lactamase that provides resistance to beta-lactam antibiotics (42). The *ampC* gene is normally repressed by AmpR when cell wall biogenesis is proceeding normally but is expressed when an elevated level of PG turnover products accumulates in the cytoplasm as a result of a beta-lactam-induced block in PG cross-linking (35–37). Thus, expression of *ampC* is tuned to respond when the balance of cell wall synthesis and degradation is upset. We therefore employed a *lacZ* reporter fused to the *ampC* promoter in *P. aeruginosa* to screen for mutants with PG homeostasis defects with the goal of identifying new factors involved in the process. The screen was successful and identified *mupP* (*PA3172*), a gene of previously unknown function, as encoding a new enzyme involved in PG recycling.

Recycling of PG turnover products in Gram-negative bacteria is carried out by one of two possible pathways, (i) the MurQ pathway used by *E. coli* and its relatives, in which the sugars of PG turnover products are funneled back into the *de novo* PG precursor synthesis pathway, or (ii) the MurU pathway, which more directly converts MurNAc from PG turnover products to UDP-MurNAc and bypasses *de novo* synthesis (Fig. 1) (33). Transposon insertion or *mupP* deletion mutant strains displayed *ampC* induction phenotypes that were identical to those of mutants defective for MurU and other members of the MurU pathway. Additionally, co-expression of *mupP* with *murU* and *amgK* was sufficient to reconstitute the MurU pathway in *E. coli*, which is normally reliant on the MurQ pathway and *de novo* synthesis. On the basis of these results, we conclude that MupP is likely to be the missing MurNAc-6P phosphatase enzyme previously predicted to be functioning in the MurU pathway (33). In support of this designation, the Mayer group has biochemically characterized MupP from *Pseudomonas putida* (44). They report in a parallel study that MupP specifically hydrolyzes MurNAc-6P to MurNAc *in vitro*. What remains unclear is why the MurU pathway converts MurNAc-6P to MurNAc before the AmgK kinase adds a phosphate back to form MurNAc-1P. In theory, the conversion of MurNAc-6P to MurNAc-1P could easily be catalyzed in a single step by a sugar phosphomutase. We therefore speculate that the less efficient pathway involving MupP and the formation of unphosphorylated MurNAc is likely to have additional physiological roles beyond PG recycling. Further studies are required to determine if and why the production of a steady-state pool of MurNAc might be beneficial for bacteria that utilize the MurU PG recycling pathway.

Mutants with the PG recycling enzyme AmpD or the PG remodeling factor DacB inactivated have previously been identified as *ampC* inducers (7, 13, 14). Defects in either enzyme are thought to promote the accumulation in the cytoplasm of anhMurNAc peptides, which convert AmpR to an activator of *ampC* expression. A blockade in PG sugar recycling by the MurU pathway has not previously been implicated in *ampC* induction or elevated beta-lactam resistance. The mechanism by which inactivation of the MurU pathway stimulates increased *ampC* expression is not known. However, it seems unlikely that the failure to recycle the MurNAc sugars would prevent proper peptide cleavage from anhMurNAc peptides by AmpD such that the inducers would accumulate appreciably to activate AmpR. Instead, we favor the idea that inhibition of the MurU pathway reduces the steady-state level of UDP-MurNAc-pep5 because of limitations in UDP-MurNAc production. Because UDP-MurNAc-pep5 competes with anhMurNAc-pep5 for binding to AmpR (35), decreased UDP-MurNAc-pep5 levels would alter the repressor/activator ratio and allow basal levels of anhMurNAc-pep5 to associate with AmpR to activate *ampC* expression and promote beta-lactam resistance. Although additional experimentation is required to test this hypothesis, the Fos hypersensitivity caused by inactivation of the MurU pathway is consistent with a defect in UDP-MurNAc production in mutant cells.

The identification of a new cell wall recycling factor by the P_*ampC*_::*lacZ* reporter screen validates the utility of this approach for uncovering novel players involved in the maintenance of cell wall homeostasis in *P. aeruginosa* and likely other Gram-negative bacteria. The screen reported here was not saturated, suggesting that additional PG biogenesis factors will be discovered upon continued screening. The identification and characterization of such factors will add to our growing understanding of the mechanisms by which bacteria build and maintain their cell wall and help us identify vulnerabilities in the process to exploit for antibiotic targeting.

## MATERIALS AND METHODS

### Media, bacterial strains, and plasmids.

*P. aeruginosa* PAO1 cells were grown in LB (1% tryptone, 0.5% yeast extract, 0.5% NaCl). When necessary, the medium was supplemented with 1 mM IPTG (isopropyl-β-d-thiogalactopyranoside), 5% sucrose, or 50 µg/ml X-Gal. For plasmid maintenance or integration, gentamicin (Gm) and Tet were used at a concentration of 50 μg/ml. For AmpC beta-lactamase induction, Fox was used at a concentration of 50 μg/ml. Unless otherwise indicated, antibiotics for viability/sensitivity assays were used at 25 (Fos), 4 (Caz), or 25 (Ctx) μg/ml.

*E. coli* cells were grown in LB. When necessary, the medium was supplemented with 100 μM IPTG. Unless otherwise indicated, the antibiotic concentrations used for *E. coli* were 25 (chloramphenicol and kanamycin), 10 (Gm), and 2 (Fos) μg/ml. The bacterial strains and plasmids used in this study are listed in Tables S1 to S3 in the supplemental material. Detailed descriptions of the strain and plasmid construction procedures can be found in Text S1.

10.1128/mBio.00102-17.2 TABLE S1  *P. aeruginosa* strains used in this study. Download TABLE S1, PDF file, 0.05 MB. Copyright © 2017 Fumeaux and Bernhardt. 2017 Fumeaux and Bernhardt This content is distributed under the terms of the Creative Commons Attribution 4.0 International license.

10.1128/mBio.00102-17.3 TABLE S2  *E. coli* strains used in this study. Download TABLE S2, PDF file, 0.1 MB. Copyright © 2017 Fumeaux and Bernhardt. 2017 Fumeaux and Bernhardt This content is distributed under the terms of the Creative Commons Attribution 4.0 International license.

10.1128/mBio.00102-17.4 TABLE S3  Plasmids used in this study. Download TABLE S3, PDF file, 0.1 MB. Copyright © 2017 Fumeaux and Bernhardt. 2017 Fumeaux and Bernhardt This content is distributed under the terms of the Creative Commons Attribution 4.0 International license.

10.1128/mBio.00102-17.1 TEXT S1  Supplemental methods and references. Download TEXT S1, PDF file, 0.2 MB. Copyright © 2017 Fumeaux and Bernhardt. 2017 Fumeaux and Bernhardt This content is distributed under the terms of the Creative Commons Attribution 4.0 International license.

### Viability assays.

For viability assays with *P. aeruginosa* or *E. coli*, overnight cell cultures were normalized to an optical density at 600 nm (OD_600_) of 0.05 and subjected to serial 10-fold dilution. Five-microliter volumes of the 10^−1^ through 10^−6^ dilutions were then spotted onto the indicated agar and incubated at 30°C (*P. aeruginosa*) or 37°C (*E. coli*) for ~24 h prior to imaging. Fos MICs was determined by the broth microdilution method. Overnight cell cultures were normalized to an OD_600_ of 0.0005 in LB and different concentrations of Fos and grown for ~24 h at 30°C. The MIC was defined as the lowest concentration that inhibited growth.

### Screening for mutants that induce *ampC* expression.

*P. aeruginosa* strain CF263 (PAO1 [P_*ampC*_::*lacZ*]) was transposon mutagenized by mating with the *E. coli* donor SM10(λpir) harboring mariner transposon delivery vector pIT2 (50). The transposon confers Tet resistance. Mating mixtures were plated on LB agar supplemented with Tet (50 µg/ml) to select for transposon mutants and nalidixic acid (25 µg/ml) to select against the *E. coli* donor. The resulting collection of colonies was resuspended in LB broth and stored at −80°C. Dilutions of the library were plated on LB containing X-Gal (40 µg/ml) to identify mutants with a constitutively active P_*ampC*_::*lacZ* reporter. The screen was not saturated, as indicated by the absence of *ampD* mutants among the isolates identified. We are therefore continuing to mine the library for additional mutants that induce the P_*ampC*_::*lacZ* reporter.

### Mapping of transposon insertion sites.

Transposon insertions were mapped by arbitrarily primed PCR (50). Transposon-chromosomal DNA junctions were amplified from mutant chromosomal DNA with primers Rnd1-PA (5′ GGCCACGCGTCGACTAGTACNNNNNNNNNNGATAT 3′) and LacZ211 (5′ TGC GGG CCT CTT CGC TAT TA 3′). The resulting PCR was used for a second PCR with primers Rnd2-PA (5′ GGCCACGCGTCGACTAGTAC 3′) and LacZ148 (5′ GGG TAA CGC CAG GGT TTT CC 3′). The final PCR product was sequenced with transposon-specific primer LacZ-124L (5′ CAG TCA CGA CGT TGT AAA ACG ACC). The transposon-chromosomal DNA junction was identified in the sequencing reads by a nucleotide BLAST search (51) against the PAO1 genome (52).

### β-Galactosidase assays.

β-Galactosidase assays were performed at room temperature. Cells from 100 μl of culture at an OD_600_ of 0.1 to 0.6 were lysed with 30 μl of chloroform and mixed with 700 μl of Z buffer (60 mM Na_2_HPO_4_, 40 mM NaH_2_PO_4_, 10 mM KCl, 1 mM MgSO_4_ heptahydrate). Each reaction mixture then received 200 μl of *o*-nitrophenyl-β-d-galactopyranoside (4 mg/ml in 0.1 M KPO_4_, pH 7.0), and the reaction was timed. When a medium yellow color developed, the reaction was stopped with 400 μl of 1 M Na_2_CO_3_. The OD_420_ of the supernatant was determined, and the units of activity were calculated with the equation U = (OD_420_ × 1,000)/[OD_660_ ⋅ time (in minutes) ⋅ volume of culture (in milliliters)].

### AmpC beta-lactamase activity assay.

AmpC activity was assessed by nitrocefin hydrolysis. Overnight bacterial cultures were subcultured 1:20 in 3 ml of LB and grown for 2 h at 30°C and 200 rpm. Cultures were split 1:1 in 2 ml of LB with or without 50 μg/ml (final concentration) Fox and incubated for an additional 1.5 h at 30°C and 200 rpm. Following incubation, 1 ml of culture was pelleted at 2,300 × *g* for 5 min, washed once with 1 ml of 50 mM sodium phosphate buffer (pH 7.0), and resuspended in 1 ml of the same cold buffer. Samples were placed on ice and lysed at 4°C by sonication with a microprobe (Q800R2; QSonica, Newtown, CT). Sonicated samples were centrifuged at 12,000 × *g* for 5 min at 4°C, and supernatants were collected. The protein concentration was determined with a Bradford assay (53) with bovine serum albumin (BSA) as the standard (G-Biosciences/Geno Technology Inc., Saint Louis, MO). Nitrocefin hydrolysis assays were performed with 96-well plates. Each reaction mixture had a final volume of 250 μl of 50 mM sodium phosphate buffer (pH 7.0) containing 10 μg of protein and 20 μg of nitrocefin (Thermo Fischer Scientific Oxoid, Waltham, MA). Nitrocefin hydrolysis was monitored by measuring the absorbance at 486 nm every 5 min for 2 h at 30°C.

### Phylogenetic analysis.

A phylogenetic tree showing the distribution of the MurU pathway proteins and MurQ in a diverse set of 1,773 bacterial taxa was constructed. The amino acid sequences of all of the members of the MurU pathway, AnmK, and MurQ were used as queries in a BLASTp search against the NCBI nonredundant database (54) with an E value cutoff of 10^−26^. A list of all of the taxa for which significant BLAST results were found was then sorted. We used a complex and diverse set of 1,773 bacterial taxa called representative genomes that is available on NCBI (ftp://ftp.ncbi.nlm.nih.gov/blast/db/, Representative_Genomes.00.tar.gz). The phylogenetic tree was constructed with PhyloT (http://phylot.biobyte.de/), and BLASTp results were plotted against the tree. The occurrence of a MupP protein is indicated by red, that of MurU is indicated by green, that of Amgk is indicated by blue, that of AnmK is indicated by purple, and that of MurQ is indicated by yellow. The tree was visualized and annotated with iToL (http://itol.embl.de/) (55).
