# Supplementary material for: Identification of MupP as a New Peptidoglycan Recycling Factor and Antibiotic Resistance Determinant in Pseudomonas aeruginosa
Source: mBio. 2017 Mar 28;8(2):e00102-17. doi: 10.1128/mBio.00102-17 (PMC5371409; doi:10.1128/mBio.00102-17)
Supplement: TABLE S1 [file mbo002173255st1.pdf]

Table S1. *Pseudomonas aeruginosa* strains used in this study.

Strain	Genotype	Source/Reference
PAO1	*Wild-type*	(1)
CF155	PAO1 Δ*dacB (PA3047)*	This study
CF612	PAO1 Δ*ampC (PA4110)*	This study
CF550	PAO1 Δ*anmK (PA0666)*	This study
CF596	PAO1 Δ*amgK (PA0596)*	This study
CF488	PAO1 Δ*murU (PA0597)*	This study
CF610	PAO1 Δ*amgK-murU (PA0596-0597)*	This study
CF592	PAO1 Δ*mupP (PA3172)*	This study
CF479	PAO1 Δ*djlA (PA0598)*	This study
CF608	PAO1 Δ*murU*Δ*ampR*	This study
CF690	PAO1 Δ*murU*Δ*ampC*	This study
CF647	PAO1 Δ*mupP*Δ*ampR*	This study
CF692	PAO1 Δ*mupP*Δ*ampC*	This study
CF521	PAO1 Δ*mupP attTn7*::P_{lac}::*empty*	This study
CF505	PAO1 Δ*mupP attTn7*::P_{lac}::*mupP*	This study
CF517	PAO1 Δ*murU attTn7*::P_{lac}::*empty*	This study
CF519	PAO1 Δ*murU attTn7*::P_{lac}::*murU*	This study
CF732	PAO1 *attTn7*::P_{lac}::*empty*	This study
CF263	PAO1 *attB*::P_{ampC} *(352 bp)-lacZ*	This study
CF268	PAO1 Δ*dacB attB*::P_{ampC} *(352 bp)-lacZ*	This study
CF316	PAO1 Δ*ampG attB*::P_{ampC} *(352 bp)-lacZ*	This study
CF312	PAO1 Δ*dacB*Δ*ampG attB*:: P_{ampC}::*lacZ*	This study
CF613	PAO1 Δ*ampC attB*::P_{ampC} *(352 bp)-lacZ*	This study
CF485	PAO1 Δ*murU attB*::P_{ampC} *(352 bp)-lacZ*	This study
CF594	PAO1 Δ*mupP attB*::P_{ampC} *(352 bp)-lacZ*	This study
CF600	PAO1 Δ*amgK attB*::P_{ampC} *(352 bp)-lacZ*	This study
CF706	PAO1 Δ*anmK attB*::P_{ampC} *(352 bp)-lacZ*	This study
CF473	PAO1 Δ*djlA attB*::P_{ampC} *(352 bp)-lacZ*	This study
