# Supplementary material for: Identification of MupP as a New Peptidoglycan Recycling Factor and Antibiotic Resistance Determinant in Pseudomonas aeruginosa
Source: mBio. 2017 Mar 28;8(2):e00102-17. doi: 10.1128/mBio.00102-17 (PMC5371409; doi:10.1128/mBio.00102-17)
Supplement: TABLE S2 [file mbo002173255st2.pdf]

Table S2. *Escherichia coli* strains used in this study.

Strain	Genotype[a]	Source/Reference[b]
DH5α	*F– hsdR17 deoR recA1 endA1 phoA supE44 thi-1 gyrA96 relA1 Δ(lacZYA-argF)U169 ϕ80dlacZΔM15*	Gibco BRL
Sm10(λpir)	*Kan^R thi-1 thr leu tonA lacY supE recA::RP4-2-Tc::Mu attλ::pir*	(2)
MG1655	*rph-1 ilvG rfb-50*	(3)
JW2421	BW25113 Δ*murQ(yfeU)::Kan^R*	(4)
CF491	MG1655 Δ*murQ(yfeU)::Kan^R*	P1(JW2421) x MG1655
CF752	MG1655 Δ*murQ(yfeU)*::*frt*	CF491/pCP20

[a] The Kan^R cassette is flanked by *frt* sites for removal by FLP recombinase. An *frt* scar remains following removal of the cassette using FLP expressed from pCP20.

[b] Strain constructions by P1 transduction are described using the shorthand: P1(donor) x recipient.
