# Supplementary material for: Identification of MupP as a New Peptidoglycan Recycling Factor and Antibiotic Resistance Determinant in Pseudomonas aeruginosa
Source: mBio. 2017 Mar 28;8(2):e00102-17. doi: 10.1128/mBio.00102-17 (PMC5371409; doi:10.1128/mBio.00102-17)
Supplement: TABLE S3 [file mbo002173255st3.pdf]

Table S3. Plasmids used in this study.

Plasmid	*Genotype*	*ori*	*Source/Reference*
pEXG2	*aacC1 sacB oriT [vector for allelic exchange]*	pBR/colE1	(5)
pFlp2	*bla sacB flp cI oriT [plasmid for Flp recombinase expression]*	pRO1600	(6) GenBank no. AF048702
pKHT103	*aacC1 bla Tn7 lacI^q P_{lac} [vector for insertion of P_{lac}-regulated sequences into the Tn7 attachment site of Pseudomonas]*	pBR/colE1	Dove lab
pTNS2	*bla oriR6K tnsABCD [plasmid for Tn7 transposase expression]*	R6K	(7)
pCP20	*bla cat flp cI857 λP_R::Rep^{TS} [plasmid for Flp recombination]*	R6K	(8)
pCF552	*aacC1 bla Tn7 lacI^q P_{lac}::mupP*	pBR/colE1	This study
pCF474	*aacC1 bla Tn7 lacI^q P_{lac}::murU*	pBR/colE1	This study
pCF436	*aacC1 bla Tn7 lacI^q P_{lac}::amgK-murU*	pBR/colE1	This study
pCF572	*aacC1 sacB oriT ‘PA3172-mupPΔ (3-223)*	pBR/colE1	This study
pCF434	*aacC1 sacB oriT ‘PA0596-amgKΔ (6-334)*	pBR/colE1	This study
pCF468	*aacC1 sacB oriT ‘PA0597-murUΔ (7-223)*	pBR/colE1	This study
pCF530	*aacC1 sacB oriT ‘PA0666-anmKΔ (5-362)*	pBR/colE1	This study
pCF462	*aacC1 sacB oriT ‘PA0598-djlAΔ (3-251)*	pBR/colE1	This study
pCF579	*aacC1 sacB oriT ‘PA4110-ampCΔ (3-289)*	pBR/colE1	This study
pCF583	*aacC1 sacB oriT ‘PA4109-ampRΔ (6-294)*	pBR/colE1	This study
pCF198	*aacC1 sacB oriT ‘PA3047-dacBΔ (1-476)*	pBR/colE1	This study

Plasmid	***Genotype***	***ori***	***Source/Reference***
pCF284	*aacC1 sacB oriT ‘PA4393-ampGΔ (1-594)*	pBR/colE1	This study
pCF826	*cat* $lacI^q$ $P_{lac::}$*::mupP*	p15A	This study
pCF836	*cat* $lacI^q$ P_{lac}*::mupP D12A*	p15A	This study
pCF758	Kan^R *lacZ -mupP (pOK12 derivative)*	p15A	This study
pCF272	Kan^R *lacZ -mupP D12A (pOK12 derivative)*	p15A	This study
pOK12	Kan^R *lacZ*	p15A	(9)
pOC1	$P_{ampC(352\ bp)}$ fused to the *lacZ* of mini-CTX-*lacZ*	pBR/colE1 (pMB1)	(10)
