# Supplementary material for: Identification of MupP as a New Peptidoglycan Recycling Factor and Antibiotic Resistance Determinant in Pseudomonas aeruginosa
Source: mBio. 2017 Mar 28;8(2):e00102-17. doi: 10.1128/mBio.00102-17 (PMC5371409; doi:10.1128/mBio.00102-17)
Supplement: TEXT S1 [file mbo002173255s1.pdf]

***E. coli* strain construction:**

The *E. coli* Δ*murQ* strain used in the reported experiments is a derivative of MG1655 (3). The mutant containing a deletion in *murQ* was constructed by P1 transduction (11) from the Keio collection strain JW2421 (4) and the removal of the Kan-resistance marker (Kan^R) flanked by *frt* sites was performed using plasmid pCP20 as described previously (12). The desired chromosomal modification was confirmed using diagnostic PCR.

Plasmid construction:

For all plasmid constructions (**Table S3**), PCR was performed using Phusion or the Q5 polymerase (New England Biolabs) according to the manufacturer's instructions. Plasmid DNA was purified using the Zippy miniprep kits (Zymo Research) while PCR fragments were purified using a Qiaquick PCR purification kit (Qiagen). Unless otherwise indicated, plasmids were constructed using the Gibson isothermal assembly method (13) and the reactions were incubated at 50°C for 30 minutes.

pCF552 [P_{TOPLAC}::*mupP*]: The construction of pCF552 for integration of an IPTG-inducible version of *mupP* at the Tn7 attachment (*att*Tn7) site, *mupP* was amplified from PAO1 genomic DNA using 5' AGC TTA GTC GAC AGC TAG CCG GAT CCC CGG GAG GAG GAT ACA TGT GAA GCG CAT GCG GCT CAA AGC GGT ACT 3' and 5' AAG GGG TTA TGC TAA AGC TTG CAT GCG GTA CTC AGC AGT CGC AGA GCG CGC GGT CGA GAA 3' primers. The resulting fragment was then inserted by Gibson isothermal assembly into a KpnI-digested expression vector pKHT103 to generate pCF552.

pCF474 [P_{TOPLAC}::*murU*]: The construction of pCF474 for integration of an IPTG-inducible version of *murU* at the Tn7 attachment (*att*Tn7) site, *murU* was amplified from PAO1 genomic DNA using 5' AGC TTA GTC GAC AGC TAG CCG GAT CCC CGG GAG GAG GAT ACA TAT GAA GGC GAT GAT CCT CGC CGC CG 3' and 5' AAG GGG TTA TGC TAA AGC TTG CAT GCG GTA CTC AGG CGT GCT CCG CCA GCA ATC GCT CGA 3' primers. The resulting fragment was then inserted by Gibson isothermal assembly into a KpnI-digested expression vector pKHT103 to generate pCF474.

pCF436 [P_{TOPLAC}::*amgK-murU*]: The construction of pCF436 for integration of an IPTG-inducible version of *amgK-murU* at the Tn7 attachment (*att*Tn7) site, the *amgK-murU* operon was amplified from PAO1 genomic DNA using 5' AGC TTA GTC GAC AGC TAG CCG GAT CCC CGG GAG GAG GAT ACA TAT GTC TGA TGA TGC CCG TTT CCA GCA GCT GA 3' and 5' AAG GGG TTA TGC TAA AGC TTG CAT GCG GTA CTC AGG CGT GCT CCG CCA GCA ATC GCT CGA CTT 3' primers. The resulting fragment was then inserted by Gibson isothermal assembly into a KpnI-digested expression vector pKHT103 to generate pCF436.

Site-directed mutagenesis was performed using the QuikChange method (Stratagene). For **pCF758 [*Kan^R lacZ -mupP* (pOK12 derivative)]**, the *mupP* gene was amplified from wild-type genomic DNA using the primers *mupP* XbaI RBS (5'-GCT ATC TAG ATG GAG AAC CCC GTA AAT GTC TGA TGA TGC CCG TTT CCA G-3') and *mupP* HindIII (5'-GCT AAA GCT TTC AGC AGT CGC AGA GCG CGC-3'). The PCR product was then digested with XbaI and HindIII and ligated into XbaI/HindIII digested pOK12. The primer sequences used for the mutagenesis are *mupP* D12A#1 (5'-AAA GCG GTA CTG TTC GCC ATG GAT GGC ACC CTG-3') and *mupP* D12A#2 (5'-CAG GGT GCC ATC CAT GGC GAA CAG TAC CGC TTT-3'). The PCR was

performed using KOD polymerase (Novagen) according to the manufacturer's instructions (65˚C annealing temperature and 2.5 minutes of extension for 20 cycles). The PCR product was directly treated for 5 hours at 37˚C with 1 μl of DpnI restriction enzyme to digest the parental double-stranded DNA. A portion of the reaction (5 μl) was used to transform chemo-competent DH5α and transformants were selected on LB plates containing 25 μg/ml kanamycin. The plasmid with the correct mutations was identified by sequencing and was designated **pCF272 [*Kan^R lacZ –mupP D12A* (pOK12 derivative)]**.

For **pCF826 [*cat $lacI^q$ P_{lac}-mupP*]** and **pCF836 [*cat $lacI^q$ P_{lac}-mupP D12A*]**, the *mupP* and *mupP D12A* genes were amplified from wild-type genomic DNA or pCF272 using the primers *mupP* XbaI RBS and *mupP* HindIII described above. The PCR products were then digested with XbaI and HindIII and ligated into XbaI/HindIII digested pDY173 [*cat $lacI^q$* $P_{lac::}$*^{ss}dsbA-envC(35-419)*] to replace the *^{ss}dsbA-envC(35-419)* gene.

P. aeruginosa strain construction.

Briefly, during *P. aeruginosa* deletion strain construction, plasmids were transferred into *P. aeruginosa* by conjugation from an *E. coli* donor [SM10(λpir)] on LB plates. Counter-selection against *E. coli* was accomplished on Vogel-Bonner minimal medium (VBMM) supplemented with 50 µg/ml gentamicin (14).

To create the Δ*mupP* strains CF592 [PAO1Δ*mupP*] and CF594 [PAO1Δ*mupP attB::*P_{ampC}*-lacZ*], pCF572 [*aacC1 sacB oriT* Δ*mupP*(3-223)] was conjugated into PAO1 [WT] and CF263 [PAO1 *attB::*P_{ampC}*-lacZ*] recipient from SM10(λpir) donor. For this purpose, PAO1 and CF263 were patched on an LB plate and grown overnight at 42°C while SM10(λpir) carrying pCF572 was

similarly grown at 37°C. Both the donor and the recipients were scraped, patched together onto an LB plate, and incubated at 37°C for ~5h. The cells were scraped, resuspended in 500 µL of VBMM, diluted 1:10, and 100 µL of the resulting suspension was plated on VBMM supplemented with 50 µg/mL gentamicin. Plates were incubated at 37°C overnight. The exconjugants were purified on LB supplemented with 50 µg/mL gentamicin. A few single colonies were allowed to grow for ~6h in plain LB broth to allow for the second plasmid recombination event, and 100 µL of the resulting culture was plated on LB supplemented with 5% sucrose to select for the loss of the plasmid-encoded *sacB* gene. Sucrose-resistant colonies were then patched onto LB plates either containing or lacking 50 µg/mL gentamicin. Gentamicin-sensitive colonies were further tested by PCR with *mupP*-flanking primers 5'- AAA GCT GGC AAC ATG AAG GAT TGG ATT CC -3' and 5'- TCC TCG CCG GTG GGG TTG TTC AGC GGG TTT -3' to confirm gene deletion. The deletion retains the first two and last four codons of the *mupP* reading frame.

To construct strains CF550 [PAO1Δ*anmK*] and CF706 [PAO1Δ*anmK attB::*P_{ampC}-*lacZ*], *anmK* was deleted from PAO1 [WT] and CF263 [PAO1 *attB::*P_{ampC}-*lacZ*] by integration and re-circularization of pCF530 [*aacC1 sacB oriT* Δ*anmK* (5-362)] as described above for CF592 construction. Sucrose-resistant, gentamicin-sensitive colonies were screened by PCR with *anmK*-flanking primers 5'- AGC TCG CTG TTC GTT GCC GGT CGT AAC G -3' and 5'- TCA GGT GGA TGG CGA CAT CCA TGC CAT CGA A -3'. The deletion retains the first four and last two codons of the *anmK* reading frame.

To construct CF596 [PAO1Δ*amgK*] and CF600 [PAO1Δ*amgK attB::*P_{ampC}-*lacZ*], *amgK* was deleted from PAO1 [WT] and CF263 [PAO1 *attB::*P_{ampC}-*lacZ*] by integration and re-

circularization of pCF434 plasmid [*aacC1 sacB oriT* Δ*amgK* (6-334)] as above. The sucrose-resistant, gentamicin-sensitive colonies were screened by PCR with *amgK*-flanking primers 5'-TGG TCG CCG ACC ACC AGC ATG CCC TTG T-3' and 5'-ACT CCA GGC GCA ACC TGC GGT CCA GCA GTT-3'. The deletion retains the first five and last five codons of the *amgK* reading frame.

To construct CF488 [PAO1Δ*murU*] and CF485 [PAO1Δ*murU attB::*P_{ampC}-*lacZ*], *murU* was deleted from PAO1 [WT] and CF263 [PAO1 *attB::*P_{ampC}-*lacZ*] by integration and re-circularization of pCF468 plasmid [*aacC1 sacB oriT* Δ*murU* (7-223)]. Sucrose-resistant, gentamicin-sensitive colonies were screened by PCR with *murU*-flanking primers 5'-AAC GGT TTC CTG CTG CTC AGT GAC CTG-3' and 5'-AGT TTG TCC GGA TGA TGC TGG CTG ATC AG-3'. The deletion retains the first six and the stop codon of the *murU* reading frame.

To construct CF155 [PAO1Δ*dacB*] and CF268 [PAO1Δ*dacB attB::*P_{ampC}-*lacZ*], *dacB* was deleted from PAO1 [WT] and CF263 [PAO1 *attB::*P_{ampC}-*lacZ*] by integration and re-circularization of pCF198 plasmid [*aacC1 sacB oriT* Δ*dacB* (1-476)]. Sucrose-resistant, gentamicin-sensitive colonies were screened by PCR with *dacB*-flanking primers 5'- CTC GAT GGC AAC TCA TAT ACT TTA AGG AAT ATT CTC A -3' and 5'- GAG GGC GGG CAG ATG TTC GCC AAT -3'. The deletion does not retain any portion of the *dacB* reading frame.

To construct CF316 [PAO1Δ*ampG attB::*P_{ampC}-*lacZ*] and CF312 [PAO1Δ*dacB* Δ*ampG attB::*P_{ampC}-*lacZ*], *ampG* was deleted from CF263 [PAO1 *attB::*P_{ampC}-*lacZ*] and CF268 [PAO1Δ*dacB attB::*P_{ampC}-*lacZ*] by integration and re-circularization of pCF284 plasmid [*aacC1 sacB oriT* Δ*ampG* (1-594)]. Sucrose-resistant, gentamicin-sensitive colonies were screened by PCR with

ampG-flanking primers 5'- TAG AGC GGT TAG AGT GCG CGT TA-3' and 5'- GTG CGA TCC ACG AAA AAG GC-3'. The deletion does not retain any *ampG* sequence.

To construct CF690 [PAO1Δ*murU*Δ*ampC*], CF692 [PAO1Δ*mupP*Δ*ampC*] and CF613 [PAO1Δ*ampC attB::*P$_{ampC}$-*lacZ*], *ampC* was deleted from CF488 [PAO1Δ*murU*], CF592 [PAO1Δ*mupP*] and CF263 [PAO1 *attB::*P$_{ampC}$-*lacZ*] by integration and re-circularization of pCF579 plasmid [*aacC1 sacB oriT* Δ*ampC* (3-289)]. Sucrose-resistant, gentamicin-sensitive colonies were screened by PCR with *ampC*-flanking primers 5'- ATG TCG ACG CGG TTG TTG TGG GTG GAC A -3' and 5'- ATG GAA ATC CTC GCC GGC ATC CGC CTC -3'. The deletion retains the 3 N-terminal and the 8 C-terminal codons of *ampC*.

To construct CF608 [PAO1Δ*murU* Δ*ampR*] and CF647 [PAO1Δ*mupP* Δ*ampR*], *ampR* was deleted from CF488 [PAO1Δ*murU*] and CF592 [PAO1Δ*mupP*] by integration and re-circularization of pCF583 plasmid [*aacC1 sacB oriT* Δ*ampR* (6-294)]. Sucrose-resistant, gentamicin-sensitive colonies were screened by PCR with *ampR*-flanking primers 5'- AAC ACT TGC TGC TCC ATG AGC CGT TCG AA -3' and 5'- AAG GTA TTC TTC TCG GCC CGC TCG AAG GT -3'. The deletion retains the 5 N-terminal and the 2 C-terminal codons of *ampR*.

To construct CF479 [PAO1Δ*djlA*] and CF473 [PAO1Δ*djlA attB::*P$_{ampC}$-*lacZ*], *djlA* was deleted from PAO1[WT] and CF263 [PAO1 *attB::*P$_{ampC}$-*lacZ*] by integration and re-circularization of pCF592 plasmid [*aacC1 sacB oriT* Δ*djlA* (3-251)]. Sucrose-resistant, gentamicin-sensitive colonies were screened by PCR with *djlA*-flanking primers 5'- TTC TTC CGT TAT CTG GAA ACC GCC GTG G -3' and 5'- AAG CAA GCC TGA CGT GAA AGG CGA AAG -3'. The deletion retains the 2 N-terminal and the 1 C-terminal codons of *djlA*.

Replicating plasmids were introduced into *P. aeruginosa* by electroporation, as described previously (15).

Construction of strain CF505 with a P_{TOPLAC}-regulated copy of *mupP* (P_{TOPLAC}::*mupP*) integrated at Tn7 locus was based on a previously described protocol (15). In brief, plasmid pCF552, which encodes a P_{TOPLAC}-regulated copy of *mupP* flanked by Tn7 transposon inverted repeats, and plasmid pTNS2, which encodes Tn7 transposase, were co-electroporated into CF592. Transformants were selected on LB plates supplemented with 50 µg/mL gentamicin. The integration of the transposon at the Tn7 attachment locus was confirmed by diagnostic PCR with PTn7R and PglmS-down primers (15). The gentamicin resistance cassette was then removed by Flp-mediated excision. Plasmid pFLP2 was electroporated and transformants were selected for growth on LB medium supplemented with 200 µg/mL carbenicillin, as described previously (15). Carbenicillin-resistant transformants were patched onto plain LB agar or LB supplemented with gentamicin to confirm the loss of the gentamycin resistance cassette. Gentamycin sensitive clones were grown overnight in liquid LB medium lacking antibiotics and purified on LB agar supplemented with 5% sucrose to select for the loss of the pFLP2 plasmid, which encodes the *sacB* gene. Isolated colonies were patched onto LB supplemented with carbenicillin or no antibiotic to confirm the loss of the pFLP2 plasmid. The construction of strain CF519 with P_{TOPLAC}::*murU* integrated at Tn7 locus was performed as above, but using plasmid pCF474.

References for supplemental materials:

1. **Stover CK**, **Pham XQ**, **Erwin AL**, **Mizoguchi SD**, **Warrener P**, **Hickey MJ**, **Brinkman FS**, **Hufnagle WO**, **Kowalik DJ**, **Lagrou M**, **Garber RL**, **Goltry L**, **Tolentino E**, **Westbrock-Wadman S**, **Yuan Y**, **Brody LL**, **Coulter SN**, **Folger KR**, **Kas A**, **Larbig K**, **Lim R**, **Smith K**, **Spencer D**, **Wong GK**, **Wu Z**, **Paulsen IT**, **Reizer J**, **Saier MH**, **Hancock RE**, **Lory S**, **Olson MV**. 2000. Complete genome sequence of Pseudomonas aeruginosa PAO1, an opportunistic pathogen. Nature **406**:959–964.

2. **Simon R**, **Priefer U**, **Pühler A**. 1983. A Broad Host Range Mobilization System for In Vivo Genetic Engineering: Transposon Mutagenesis in Gram Negative Bacteria. Nat Biotechnol **1**:784–791.

3. **Guyer MS**, **Reed RR**, **Steitz JA**, **Low KB**. 1981. Identification of a sex-factor-affinity site in E. coli as gamma delta. Cold Spring Harb Symp Quant Biol **45 Pt 1**:135–140.

4. **Baba T**, **Ara T**, **Hasegawa M**, **Takai Y**, **Okumura Y**, **Baba M**, **Datsenko KA**, **Tomita M**, **Wanner BL**, **Mori H**. 2006. Construction of Escherichia coli K-12 in-frame, single-gene knockout mutants: the Keio collection. Mol Syst Biol **2**:2006.0008.

5. **Rietsch A**, **Vallet-Gely I**, **Dove SL**, **Mekalanos JJ**. 2005. ExsE, a secreted regulator of type III secretion genes in Pseudomonas aeruginosa. PNAS **102**:8006–8011.

6. **Hoang TT**, **Karkhoff-Schweizer RR**, **Kutchma AJ**, **Schweizer HP**. 1998. A broad-host-range Flp-FRT recombination system for site-specific excision of chromosomally-located DNA sequences: application for isolation of unmarked Pseudomonas aeruginosa mutants. Gene **212**:77–86.

7. **Choi K-H**, **Gaynor JB**, **White KG**, **Lopez C**, **Bosio CM**, **Karkhoff-Schweizer RR**, **Schweizer HP**. 2005. A Tn7-based broad-range bacterial cloning and expression system. Nature Methods **2**:443–448.

8. **Cherepanov PP**, **Wackernagel W**. 1995. Gene disruption in Escherichia coli: TcR and KmR cassettes with the option of Flp-catalyzed excision of the antibiotic-resistance determinant. Gene **158**:9–14.

9. **Vieira J**, **Messing J**. 1991. New pUC-derived cloning vectors with different selectable markers and DNA replication origins. Gene **100**:189–194.

10. **Caille O**, **Zincke D**, **Merighi M**, **Balasubramanian D**, **Kumari H**, **Kong K-F**, **Silva-Herzog E**, **Narasimhan G**, **Schneper L**, **Lory S**, **Mathee K**. 2014. Structural and functional characterization of Pseudomonas aeruginosa global regulator AmpR. J Bacteriol **196**:3890–3902.

11. **Miller JH**. 1972. Experiments in molecular genetics.

12. **Datsenko KA**, **Wanner BL**. 2000. One-step inactivation of chromosomal genes in Escherichia coli K-12 using PCR products. PNAS **97**:6640–6645.

13. **Gibson DG**, **Young L**, **Chuang R-Y**, **Venter JC**, **Hutchison CA**, **Smith HO**. 2009. Enzymatic assembly of DNA molecules up to several hundred kilobases. Nature Methods **6**:343–345.

14. **Vogel HJ**, **Bonner DM**. 1956. Acetylornithinase of Escherichia coli: partial purification and some properties. J Biol Chem **218**:97–106.

15. **Choi K-H**, **Schweizer HP**. 2006. mini-Tn7 insertion in bacteria with single attTn7 sites: example Pseudomonas aeruginosa. Nat Protoc **1**:153–161.
